# Supplementary material for: Digital plasmonic nanobubble detection for rapid and ultrasensitive virus diagnostics
Source: Nat Commun. 2022 Mar 30;13:1687. doi: 10.1038/s41467-022-29025-w (PMC8967834; doi:10.1038/s41467-022-29025-w)
Supplement: Supplementary file 1 — Supplementary Information [file 41467_2022_29025_MOESM1_ESM.docx]

Supplementary Materials for

**Digital Plasmonic Nanobubble Detection for Rapid and Ultrasensitive** **Virus Diagnostics**

Yaning Liu^1, 7^, Haihang Ye^1, 7, *^, HoangDinh Huynh^2^, Chen Xie^1^, Peiyuan Kang^1^, Jeffrey S. Kahn^2, 3^, Zhenpeng Qin^1, 4, 5, 6, *^

*^1^Department of Mechanical Engineering, University of Texas at Dallas, Richardson, Texas 75080, United States*

*^2^Departments of Pediatrics, University of Texas Southwestern Medical Center, 5323 Harry Hines Boulevard, Dallas, Texas 75390, United States*

*^3^Departments of Microbiology, University of Texas Southwestern Medical Center, 5323 Harry Hines Boulevard, Dallas, Texas 75390, United States*

*^4^Department of Surgery, University of Texas Southwestern Medical Center, 5323 Harry Lines Blvd, Dallas, Texas 75390, United States*

*^5^Department of Bioengineering, University of Texas at Dallas, Richardson, Texas 75080, United States*

*^6^Center for Advanced Pain Studies, University of Texas at Dallas, Richardson, Texas 75080, United States*

^7^These authors contributed equally

^*^Corresponding authors E-mail: Haihang.Ye@utdallas.edu; Zhenpeng.Qin@utdallas.edu

**Content of Supporting Information**

**Table S1.** Comparison of various digital assays

**Fig. S1.** DIAMOND apparatus and laser beam characterization

**Fig. S2.** Morphologies and size distribution histograms of different AuNPs by TEM

**Fig. S3.** Optical characterization of AuNPs with different sizes

**Table S2.** Summary of the characterization of AuNPs used in the study

**Fig. S4.** Probability distribution histograms of the AuNPs number (k) in each virtual compartment for a given λ, based on Poisson statistics

**Fig. S5.** The schematic illustration of a typical PNB signal recorded by a photodetector (PD)

**Fig. S6.** Colorimetric detection of the homogeneous assay using AuNPs as probes and SiO_2_ beads as targets

**Fig. S7.** Preparation and characterization of AuNP-based probes for purified RSV detection

**Fig. S8.** Absorbance spectra monitoring for the homogeneous immunoassays of different respiratory viruses with varied titers (PFU/mL)

**Fig. S9.** RSV detection using a commercial lateral flow assay kit (BinaxNOW, Abbott)

**Fig. S10.** Representative PNB signal traces (100 pulses) for the assay solutions that incubating Au-Synagis probes with serial dilutions of RSV suspensions

**Fig. S11.** Representative PNB signal traces (100 pulses) for the assay solutions that incubating Au-Synagis probes with suspensions of different respiratory viruses.

**Fig. S12.** Preparation and characterization of bovine serum albumin (BSA)-backfilled AuNP-Synagis probes for RSV detection

**Fig. S13.** Representative PNB signal traces (100 pulses) for the assay solutions that incubating BSA-backfilled Au-Synagis probes with different viruses spiked in the nasal swab samples.

**Fig. S14.** Detection of viruses spiked in the nasal swab samples by DIAMOND using Au-Synagis probes.

**Fig. S15.** Representative PNB signal traces (100 pulses) for the assay solutions that incubating BSA-backfilled Au-Synagis probes with RSV of different titers that were spiked in nasal swab samples

**Table S3.** Primer sequences for loop-mediated isothermal amplification of RSV strain A2 RNA

**Fig. S16.** Detection of RSV RNA via digital loop-mediated isothermal amplification (dLAMP)

**Fig. S17.** Detection of RNA extracts from RSV spiked samples via dLAMP

**Fig. S18.** Design of benchtop device integrating DIAMOND

**Fig. S19.** Detection of 75 nm AuNPs by DIAMOND using a nanosecond laser (Wedge-HB-532, RPMC)

**Fig. S20.** The schematic illustrates the calculation of sampling efficiency for the probe beam based on the current setup

**Fig. S21.** The detection performance for the detection of 75 nm AuNPs (λ=0.0004) by DIAMOND with increasing counting number

**Table S4.** A prediction on the sensitivity enhancement by increasing the counting number for DIAMOND

**Fig. S22.** Example of the PNB generation probability curve of 15 nm AuNPs used to determine the laser fluence threshold

**Fig. S23.** Data sorting by a gating method

**Table S5.** One-pot recipe for the synthesis of AuNPs based on the seed-growth method

**Fig. S24.** Large scale preparation of RSV A2

**Fig. S25.** Endpoint dilution assay for RSV quantification

**Table S1. Comparison of various digital assays.**

| **Digital assays** | **Sensing format** | **Sample partition** | **Label** | **Signal amplification** | **Readout** | **Ref** |
| --- | --- | --- | --- | --- | --- | --- |
| **Erenna** | ^c^Bead-based ELISA | Laser confocal/Capillary electrophoresis | Fluorescence | No | Counting | 3 |
| **^a^dELISA (SIMOA)** | Bead-based ELISA | Microwells array | Enzyme | Yes | Imaging | 4 |
| **^b^dLAMP** | ^d^LAMP | Droplets | Nucleic acids | Yes | Counting | 34 |
|  | LAMP | Microwells array | Nucleic acids | Yes | Counting | 35, 36 |
| **Microscopy** | Bead-based ELISA + Isothermal amplificaiton | Individual isolates | Magnetic beads | Yes | Imaging | 5 |
|  | ^e^Homogeneous assay | Microwells array | Magnetic beads | No | Particle motion | 17, 18 |
|  |  | Individual isolates |  |  |  | 19 |
|  | Homogeneous assay | Individual isolates | Plasmonic NPs or Quantum dots | No | Imaging | 14, 16 |
|  | ^f^Heterogeneous sandwich assay |  |  |  |  | 11-13, 15 |
| **DIAMOND** | Homogeneous assay | Laser confocal/ Capillary | Plasmonic NPs | No | Counting | This work |

^a^: dELISA is digital enzyme-linked immunosorbent assay.

^b^: dLAMP is digital loop-mediated isothermal amplification. Similar work includes digital polymerase chain reaction and digital clustered regularly interspaced short palindromic repeats-based detection.

^c^: Bead-based ELISA means performing ELISA on individual beads. Typical procedures include conjugation of primary capture antibody on beads, linkage of enzyme on a secondary antibody, and incubation of antigen with both antibodies. Multiple cycles of washing steps are required during each step to remove the impurities. Amplification is also needed.

^d^: LAMP format means the target needs to be amplified prior to the measurement. Primers and temperature control are needed for specific amplification.

^e^: Homogeneous assay means using one type of labels to conjugate a single target, followed by measuring without additional steps.

^f^: Heterogeneous sandwich assay sensing format means using two types of labels to conjugate a single target with one label pre-immobilized on a surface, followed by washing steps to remove the other label in free and then measuring the signals.

**
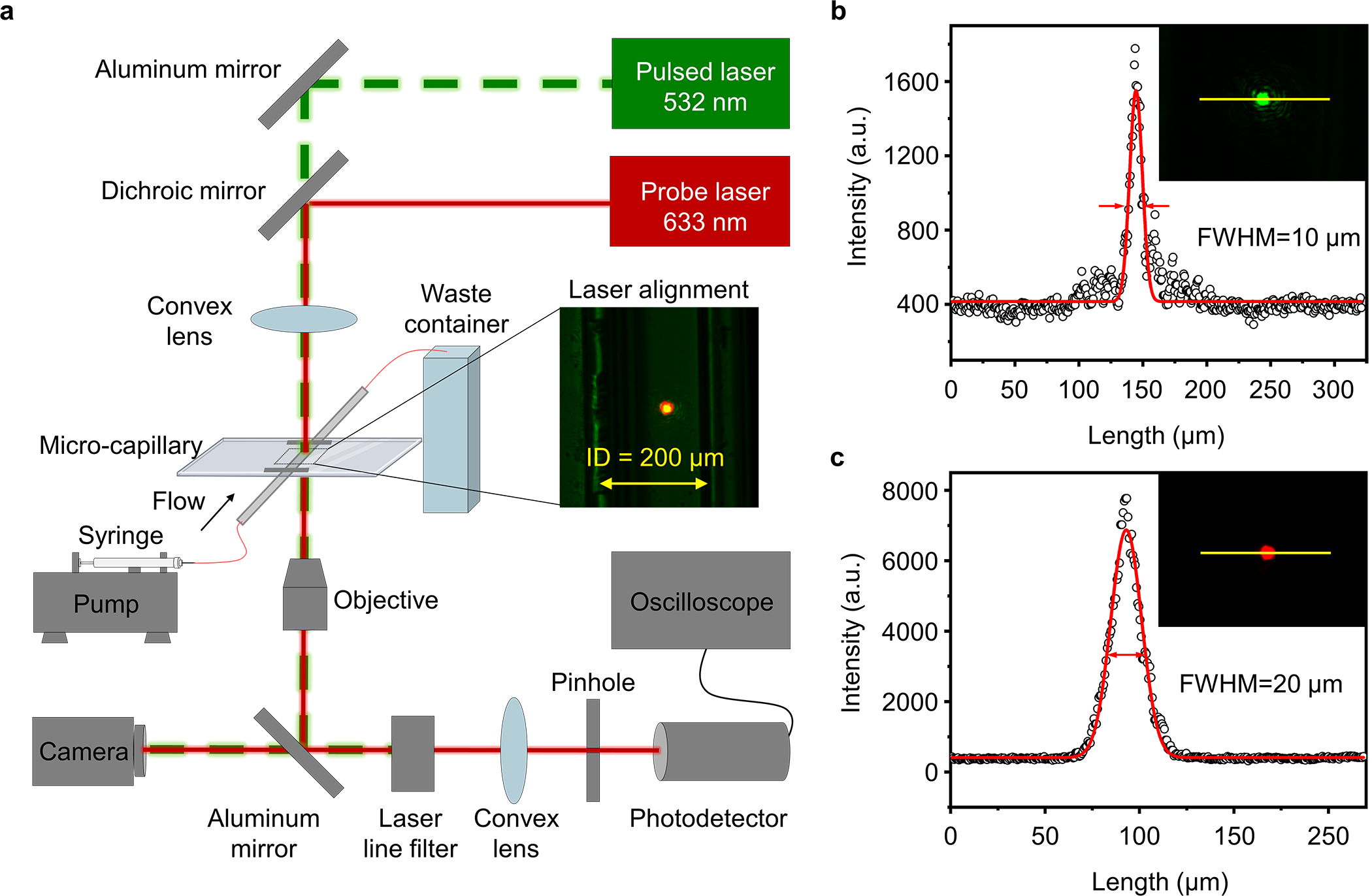
**

Fig. S1. DIAMOND apparatus and laser beam characterization. (a) The schematic illustration of the experimental setup. The intensity profiles of (b) pump and (c) probe laser beams. The scatters were fitted with a normal distribution, where the full width at half maximum (FWHM) was used as beam diameters.

Note: 1) for the volume of detection zone, it can be calculated as: $\boldsymbol{V= \pi}\boldsymbol{R}^{\boldsymbol{2}}\boldsymbol{l=\pi}\boldsymbol{\times}\boldsymbol{(5}{\boldsymbol{\mu m}\boldsymbol{)}}^{\boldsymbol{2}}\boldsymbol{\times}\boldsymbol{200}\boldsymbol{\mu m}\boldsymbol{=16 pL}$; 2) At 50 Hz laser pulse, the laser scanning speed = beam diameter × frequency = 20 µm × 50 Hz = 1,000 µm/s. The injection speed of syringe pump is set to be 6 µL/min. Given the inner diameter of cubic micro-capillary is 200 µm, the flow speed = injection speed / cross-section area of capillary = 6 µL/min / (200 µm)^2^ = 2,500 µm/s. The flow speed is faster than the laser scanning speed and makes the non-overlapping detection of PNB signals.


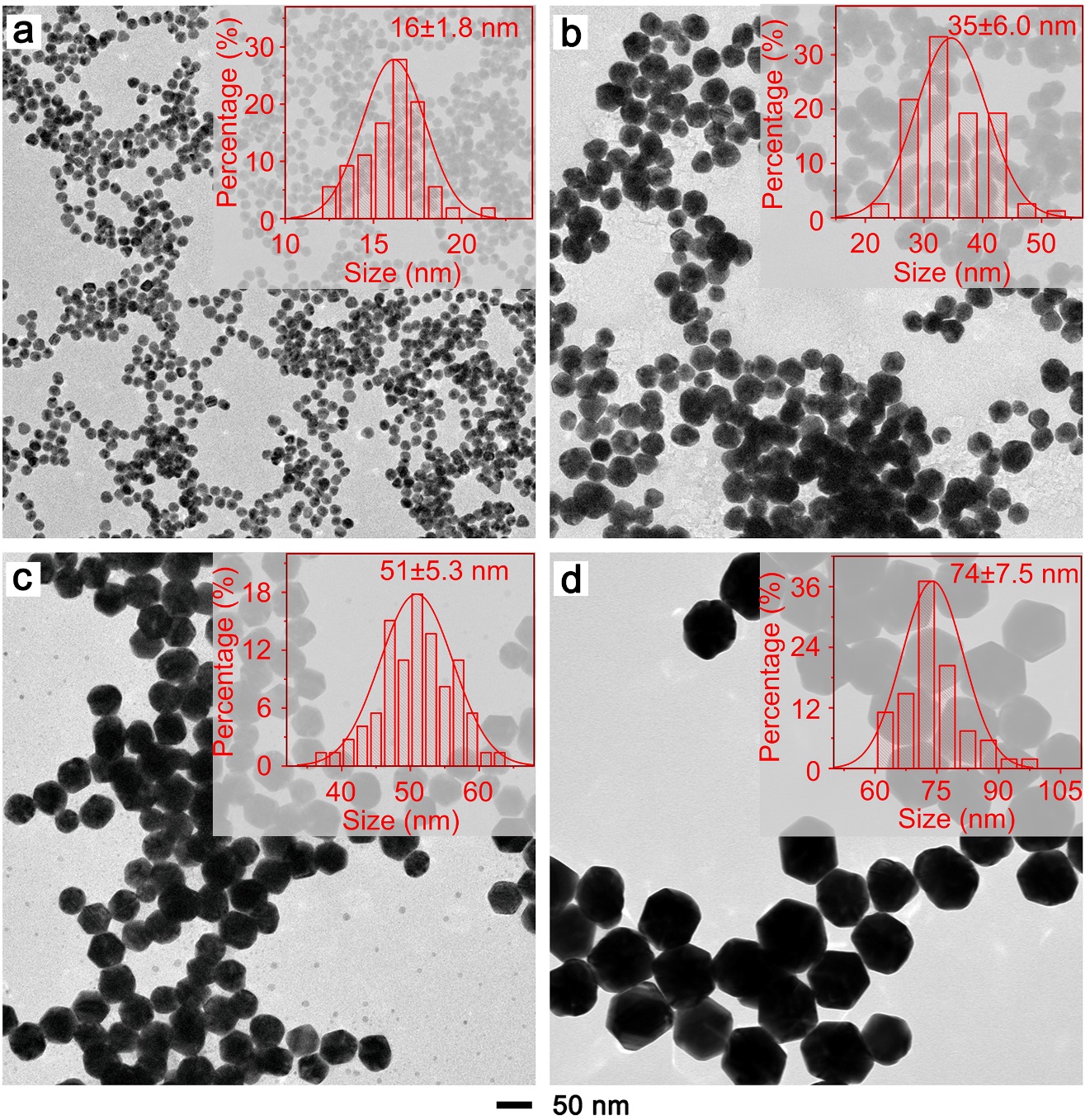


Fig. S2. Morphologies and size distribution histograms of different AuNPs by TEM. The results were counted by randomly measuring 200 particles in TEM images. For simplicity, we referred to the AuNPs in (a-d) as 15 nm, 35 nm, 50 nm, and 75 nm, respectively, and used them throughout the whole manuscript.


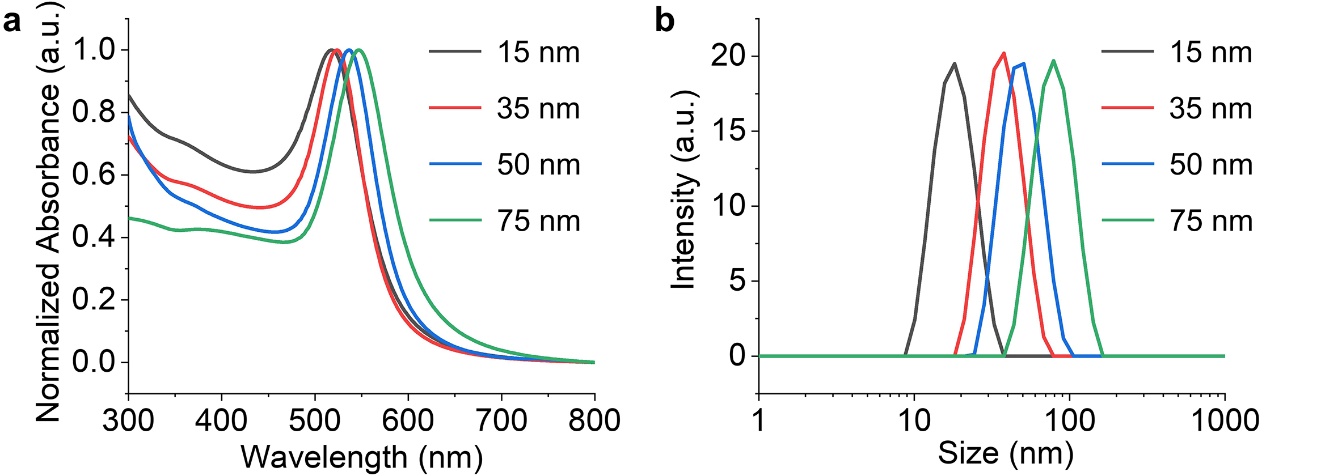


Fig. S3. Optical characterizations of AuNPs with different sizes. (a) UV-Vis measurements. (b) dynamic light scattering (DLS) analysis.

Table S2. Summary of the characterization of AuNPs used in the study.

| AuNP size (nm) | TEM | | UV-Vis spectra | DLS analysis | |
| --- | --- | --- | --- | --- | --- |
|  | ^a^Diameter (nm) | Standard deviation (nm) | Peak location (nm) | Diameter (nm) | Polydispersity index (PDI) |
| 15 | 16 | 1.8 | 518 | 17.3 | 0.062 |
| 35 | 35 | 6.0 | 524 | 38.1 | 0.062 |
| 50 | 51 | 5.3 | 537 | 55.2 | 0.067 |
| 75 | 74 | 7.5 | 547 | 76.2 | 0.044 |

^a^: Measured by counting 200 nanoparticles randomly.


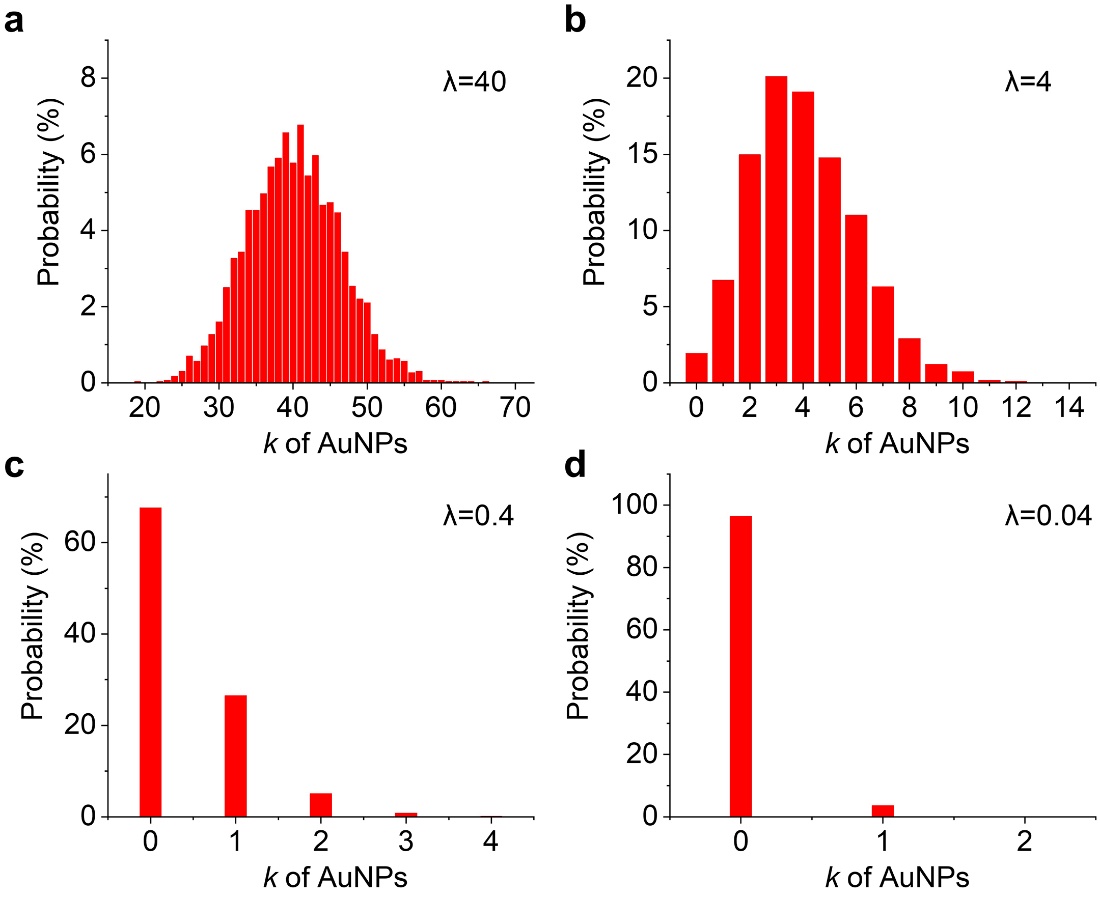


Fig. S4. Probability distribution histograms of the AuNPs number (*k*) in each virtual compartment for a given λ, based on Poisson statistics.


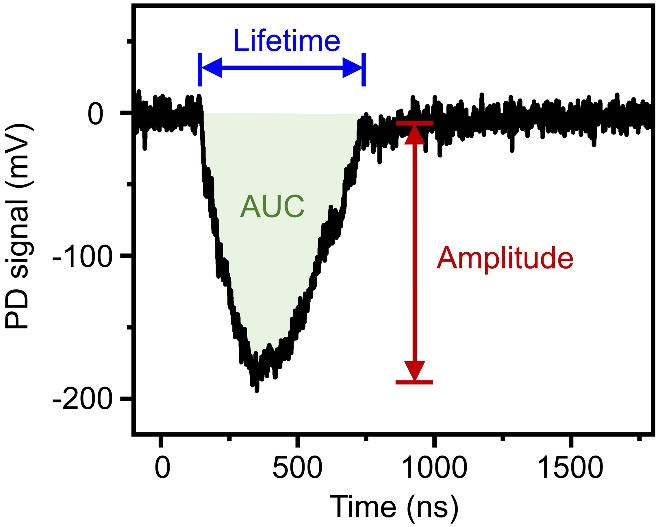


Fig. S5. The schematic illustration of a typical PNB signal recorded by a photodetector (PD). The signal includes the amplitude (peak intensity), lifetime (peak width), and area-under-curve (AUC, peak area). AUC is the integral value of amplitude along the lifetime. In the present work, only values of amplitude and AUC were used as indexes.


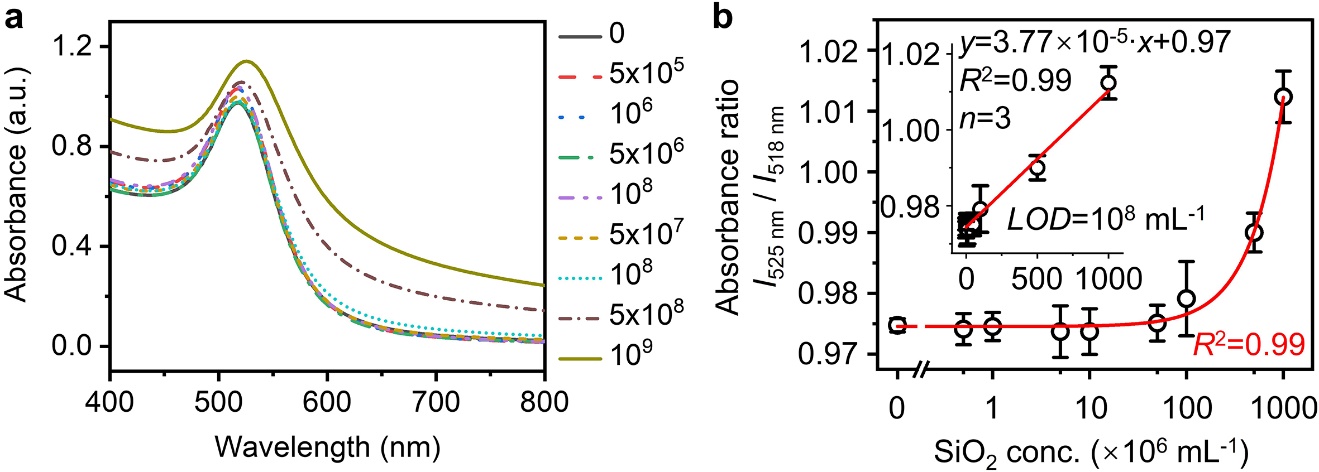


Fig. S6. Colorimetric detection of the homogeneous assay using AuNPs as probes and SiO_2_ beads as targets. (a) Absorbance spectra of assay solutions with different SiO_2_ beads concentrations (particles/mL). (b) Analysis of the colorimetric detection result. Inset shows the linear range of the colorimetric detection. Error bars indicate the standard deviations of three independent measurements, and the *LOD* was calculated as 3 standard deviations of the control divided by the slope of the regression line.


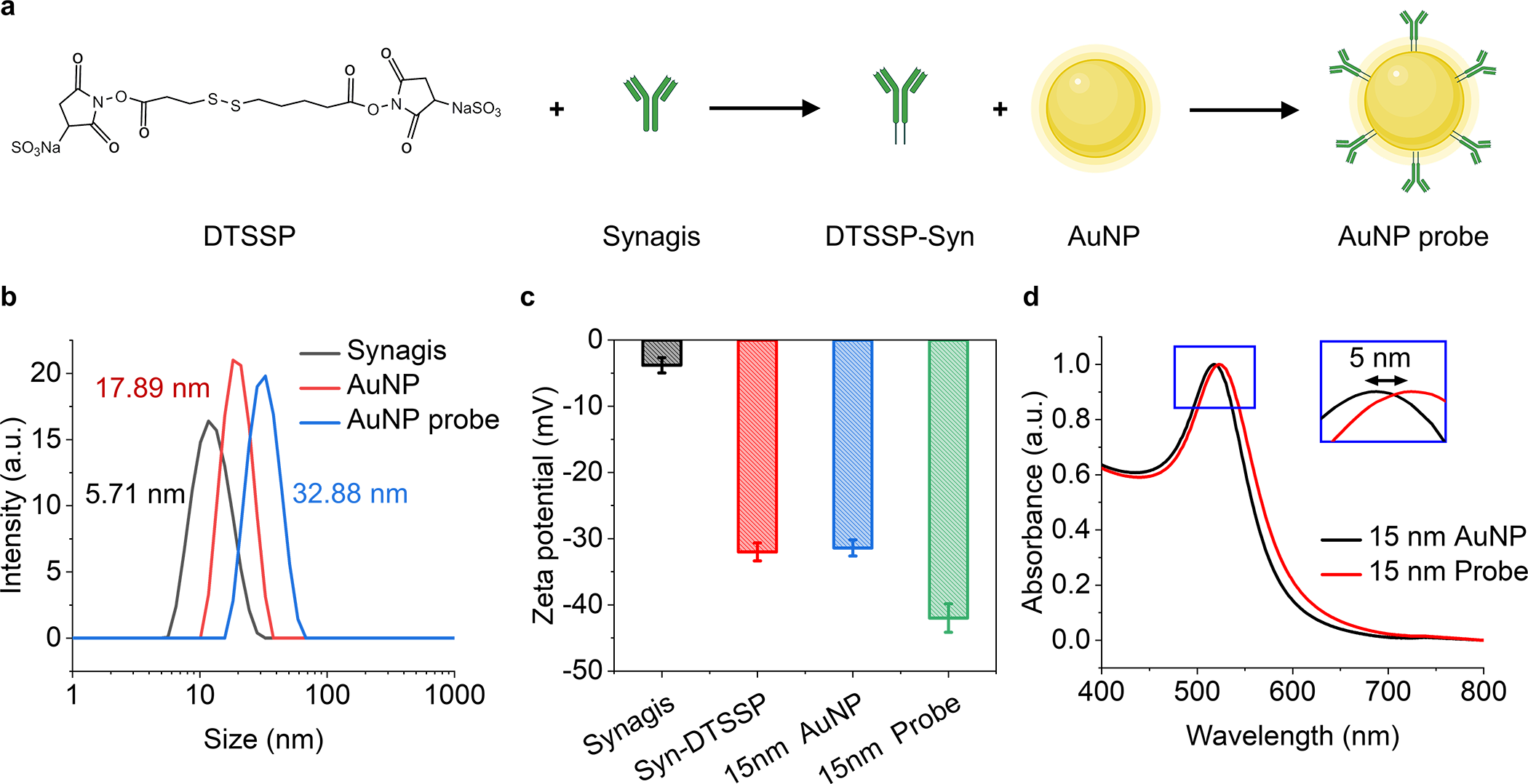


Fig. S7. Preparation and characterization of AuNP-based probes for purified RSV detection. (a) The schematic illustrates the preparation of 15 nm AuNP probes. (b) DLS, (c) Zeta potential, and (d) UV-Vis measurements for the characterization of the AuNPs before and after conjugating with antibody-linked DTSSP.


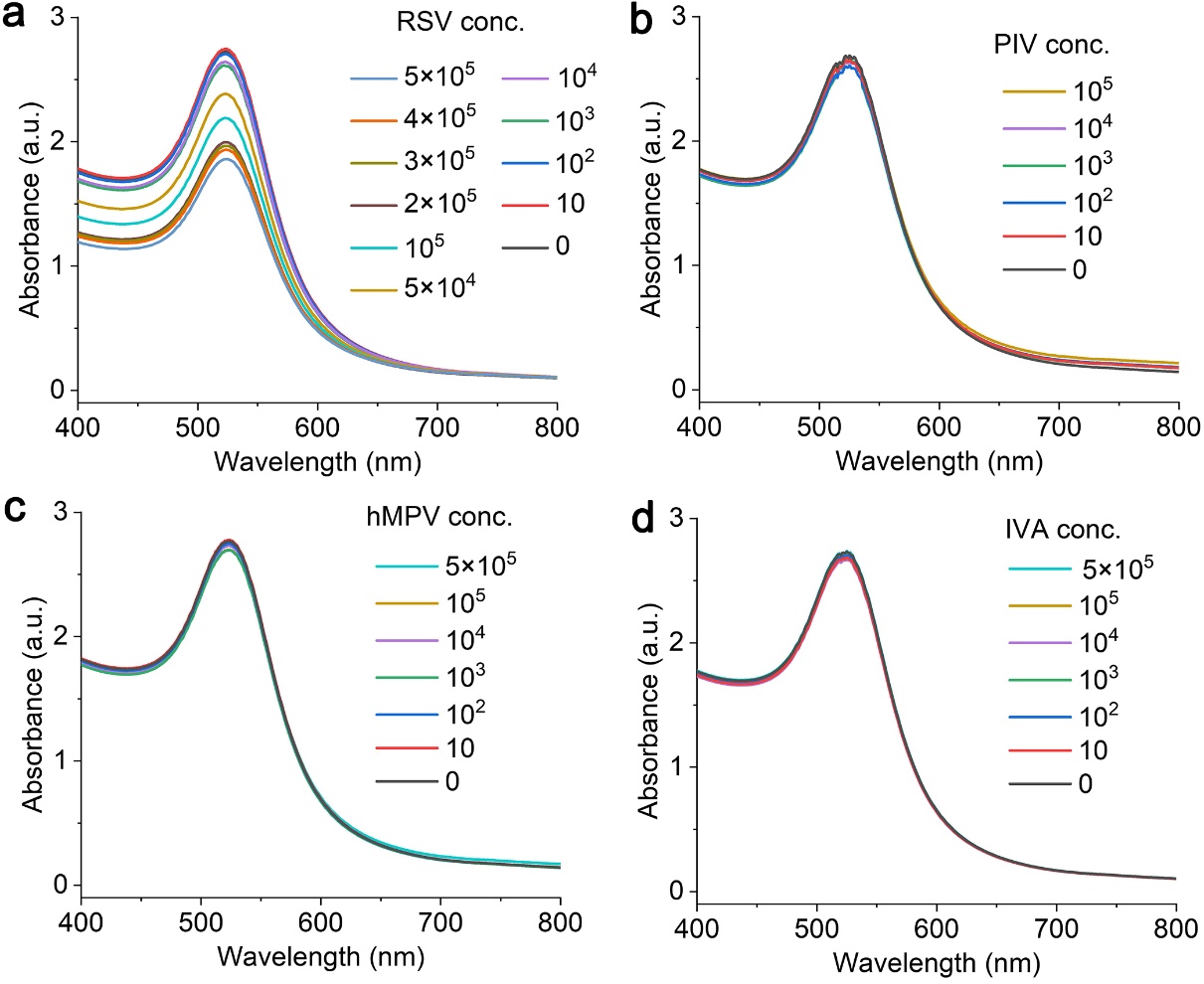


Fig. S8. Absorbance spectra monitoring for the homogeneous immunoassays of different respiratory viruses with varied titers (PFU/mL). (a) RSV=respiratory syncytial virus. (b) PIV=Parainfluenza viruses. (c) IVA=Influenza A. (d) hMPV=Human metapneumovirus.


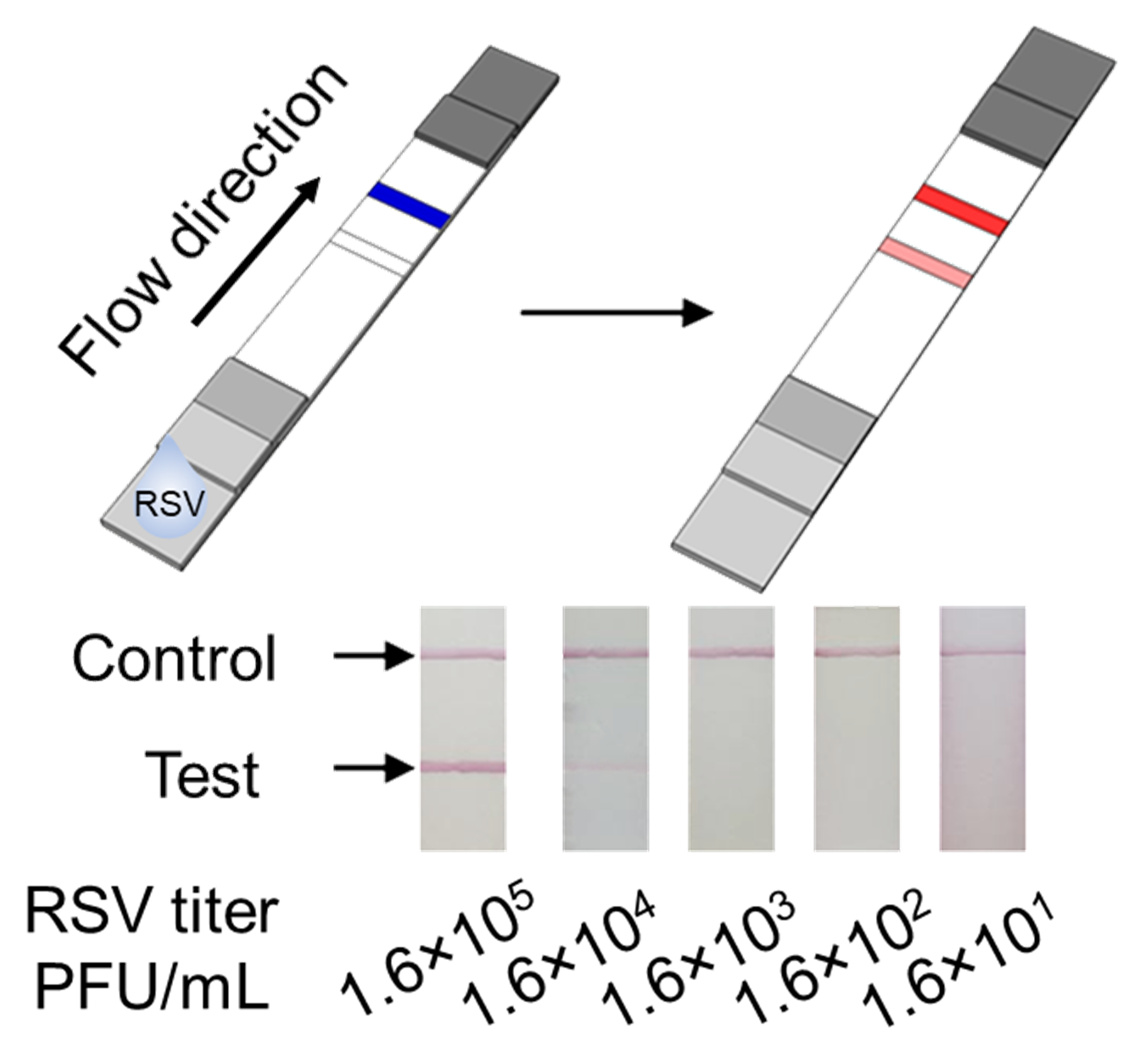


Fig. S9. RSV detection using a commercial lateral flow assay kit (BinaxNOW, Abbott). The schematic illustrates the assay operation and the digital photographs show the corresponding detection results for different RSV titers. The detection limit was estimated to be 1.6×10^4^ PFU/mL.

**
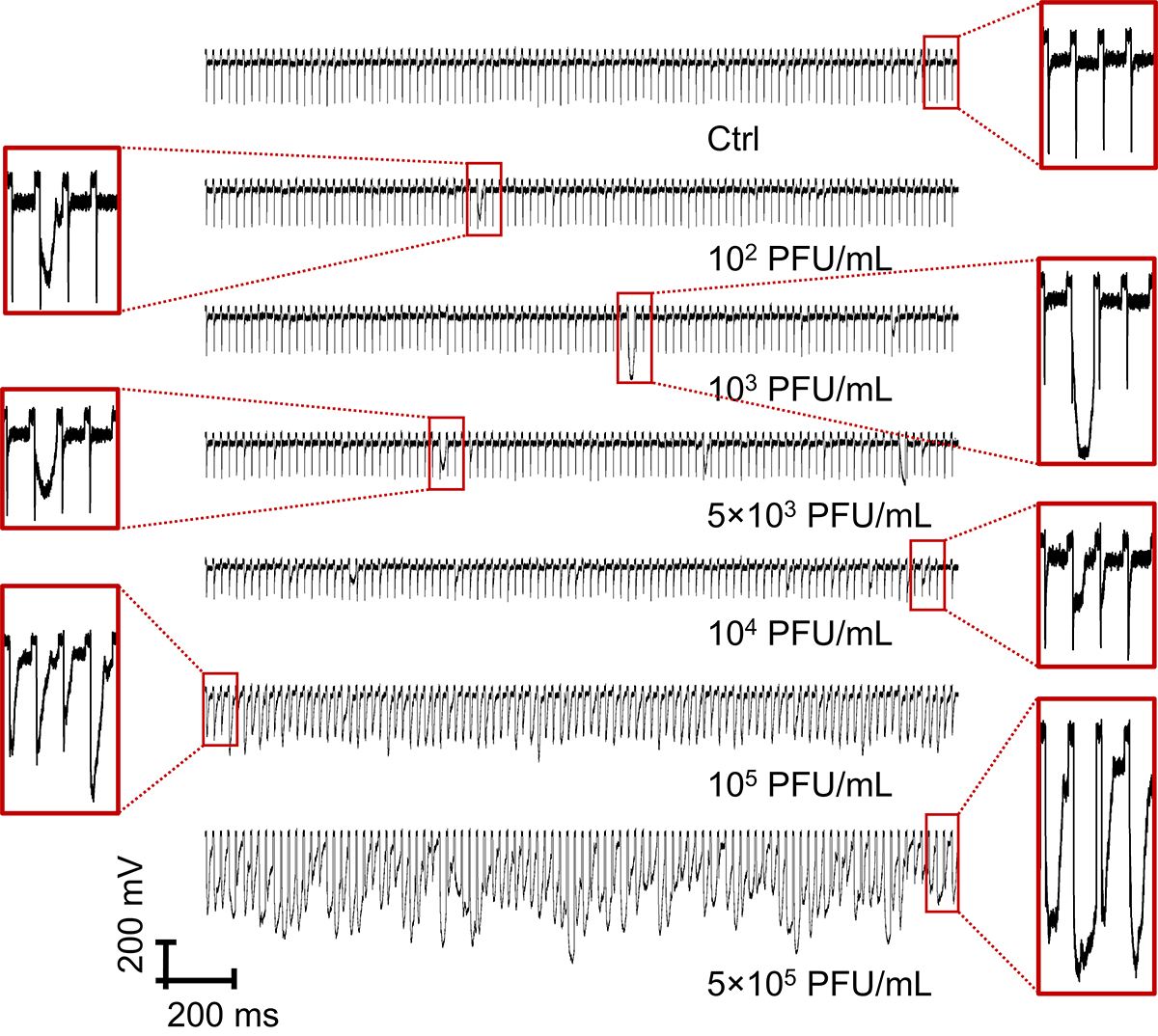
**

Fig. S10. Representative PNB signal traces (100 pulses) for the assay solutions that incubating Au-Synagis probes with serial dilutions of RSV suspensions. The RSV is dispersed in 2 mM borate buffer.


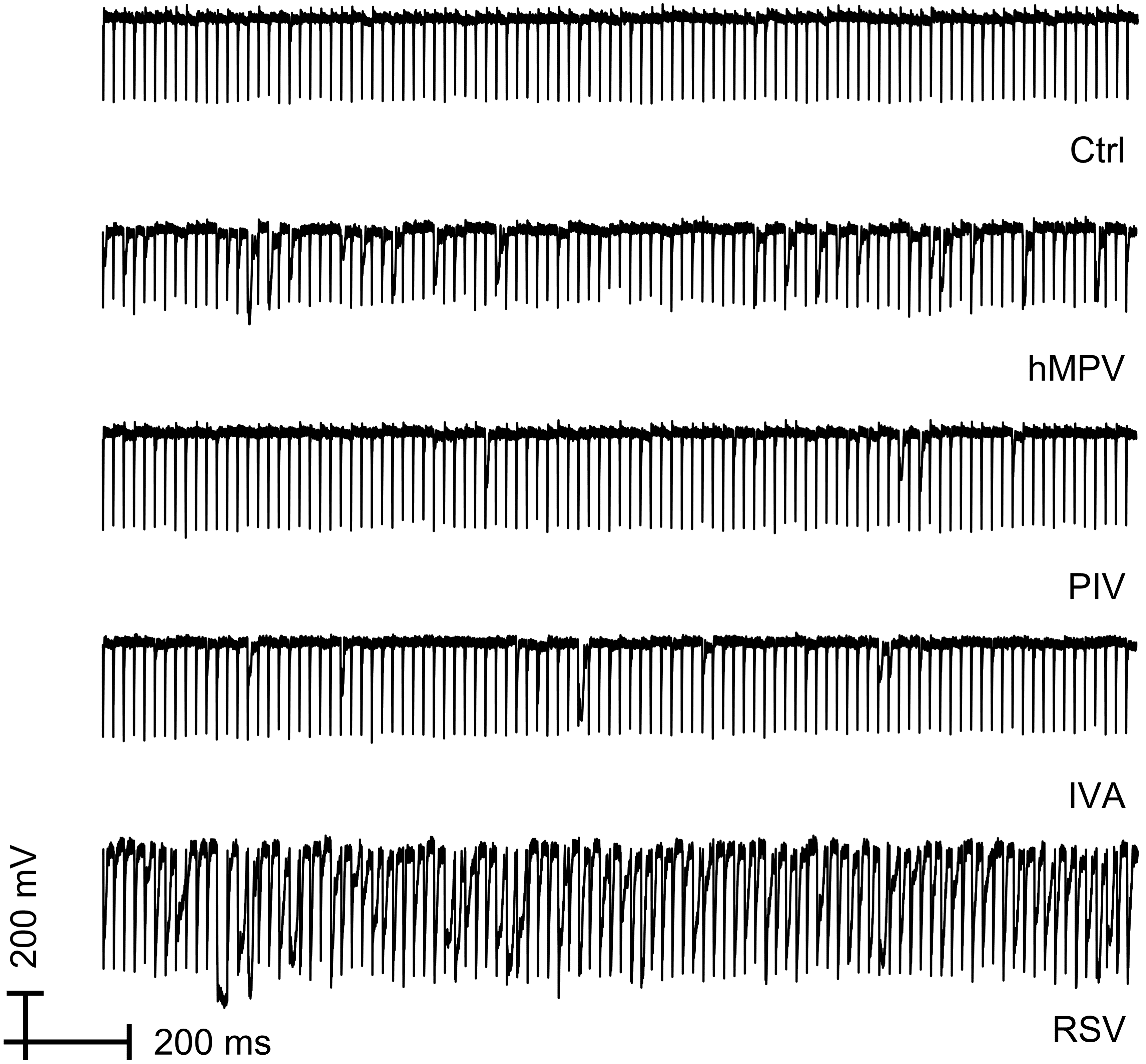


Fig. S11. Representative PNB signal traces (100 pulses) for the assay solutions that incubating Au-Synagis probes with suspensions of different respiratory viruses. The Ctrl is 2 mM borate buffer used as a control. hMPV is Human metapneumovirus, PIV is Parainfluenza viruses, and IVA is Influenza viruses A. Those control viruses are used as received without purification. The concentration of all viruses was kept the same as 10^5^ PFU/mL in borate buffer.

Note: The as-received control viruses are dispersed in cell culture fluid that contains impurities like additives and cell debris that may cause the non-specific aggregation of Au probes.


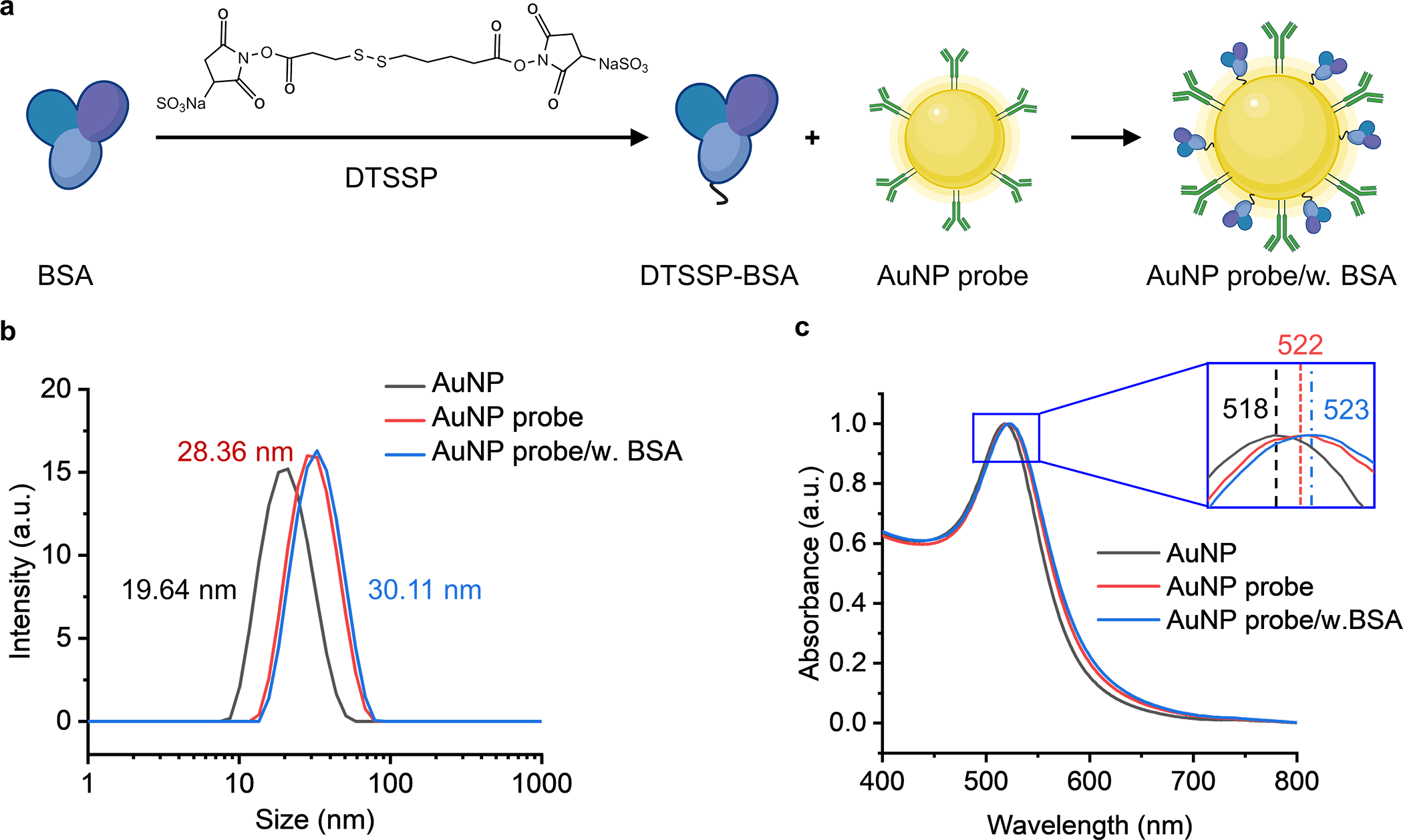


Fig. S12. Preparation and characterization of bovine serum albumin (BSA)-backfilled AuNP-Synagis probes for unpurified virus detection. (a) Schematic illustration of the BSA- backfilled AuNP probes preparation via DTSSP cross-linking. (b) DLS and (c) UV-Vis measurement for the AuNP probes.


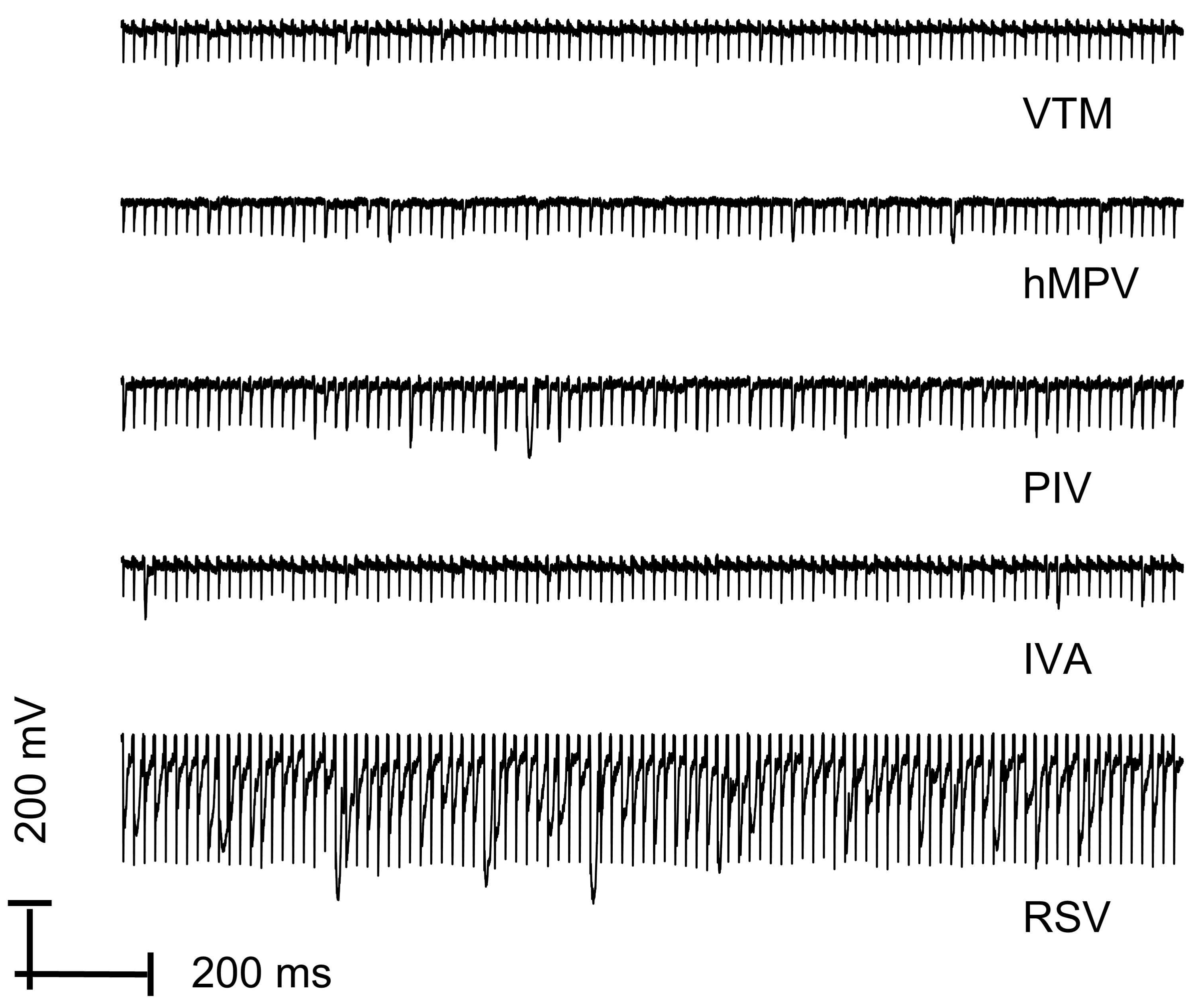


Fig. S13. Representative PNB signal traces (100 pulses) for the assay solutions that incubating BSA-backfilled Au-Synagis probes with different viruses spiked in the nasal swab samples. The concentration of all viruses was kept the same as 10^5^ PFU/mL. VTM is a viral transport medium.


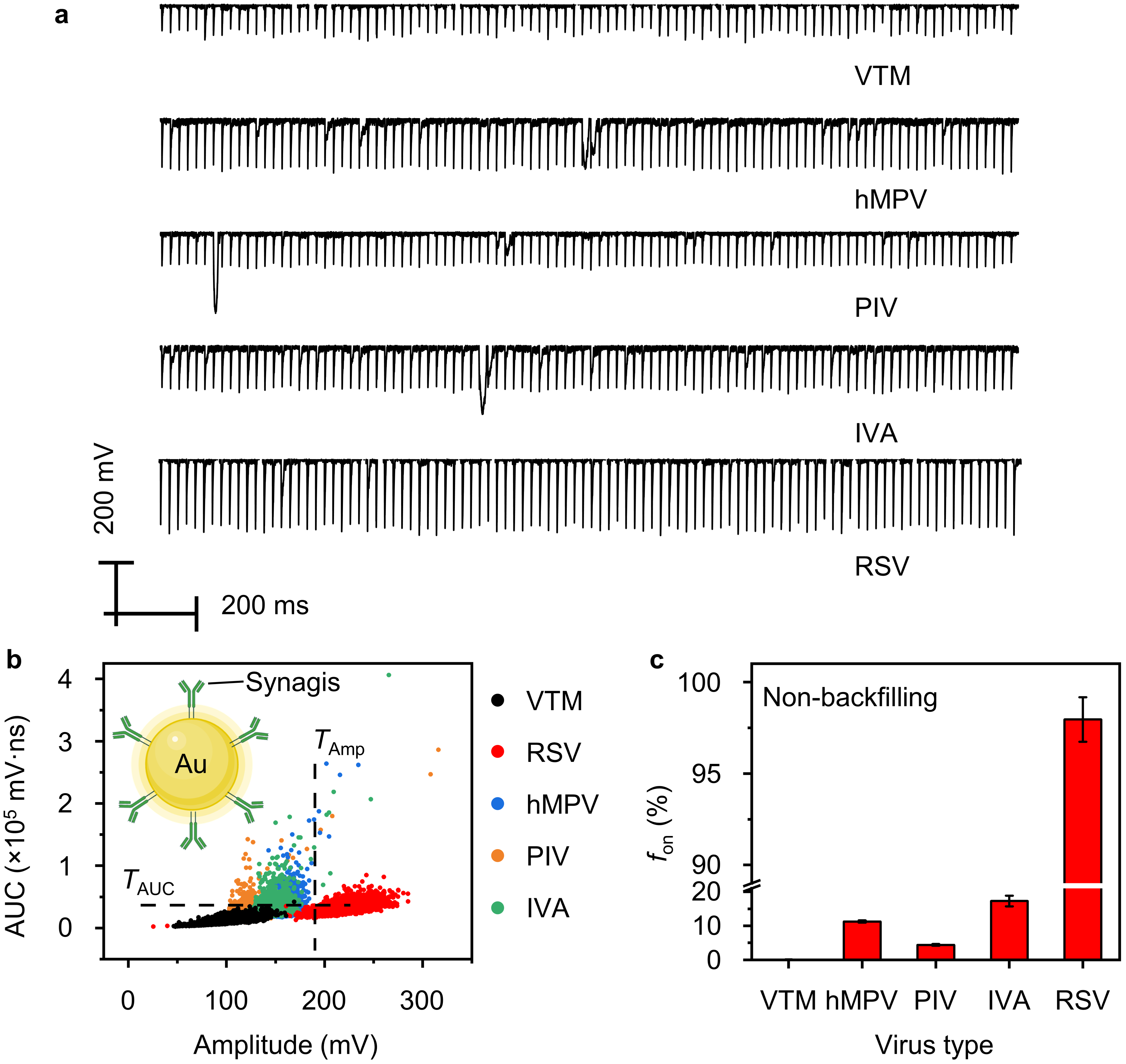


Fig. S14. Detection of viruses spiked in the nasal swab samples by DIAMOND using Au-Synagis probes. (a) Representative PNB signal traces (100 pulses) for the assay solutions that incubating Au-Synagis probes with different viruses. The concentration of all viruses was kept the same as 10^5^ PFU/mL. (b) Bivariate plot of amplitude and AUC extracted from 3,000 pulses for the assay solutions. Dashed lines indicate the thresholds calculated from the negative control sample. (c) Corresponding *f*_on_ counted from (b) against different respiratory viruses.


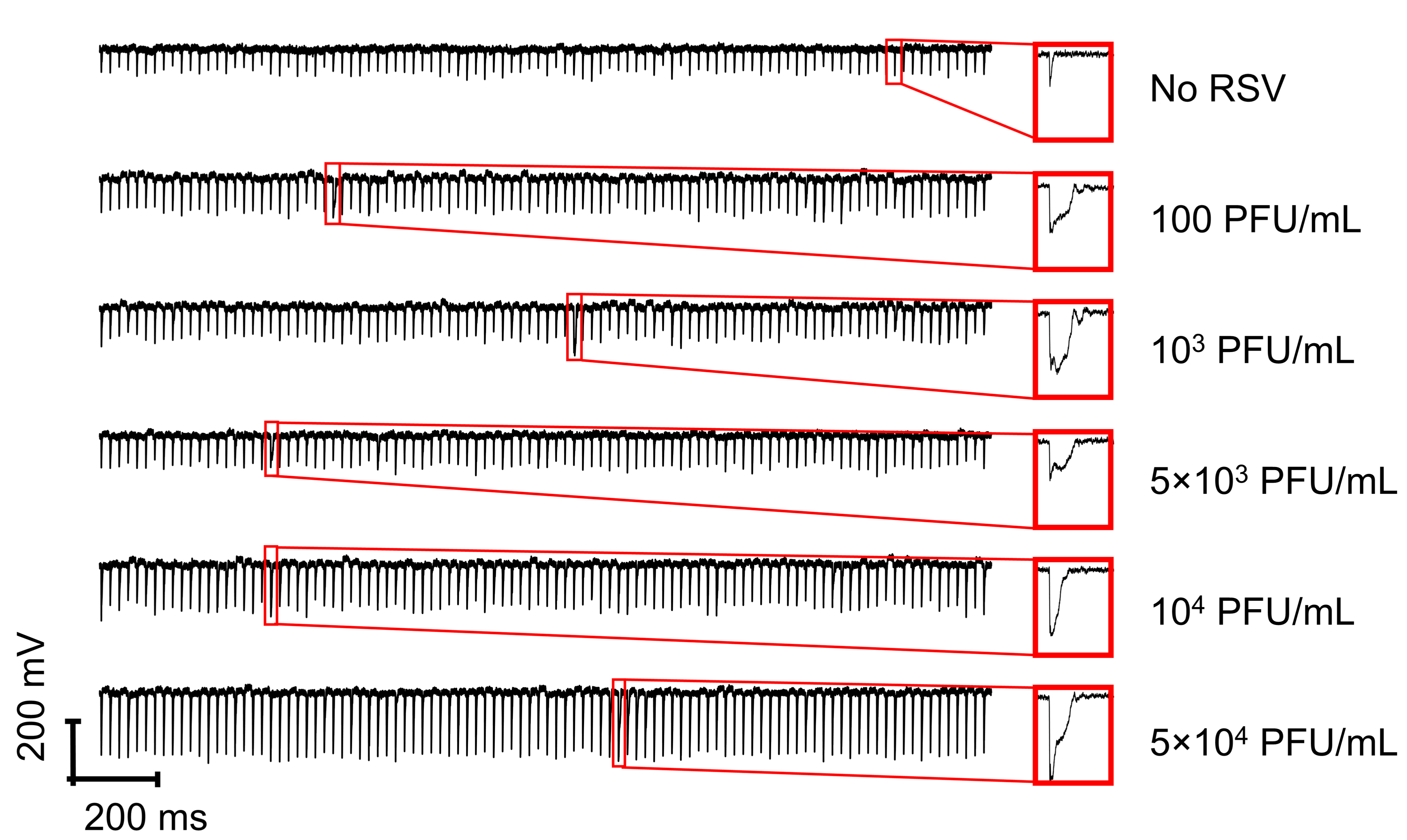


Fig. S15. Representative PNB signal traces (100 pulses) for the assay solutions that incubating BSA-backfilled Au-Synagis probes with RSV of different titers that were spiked in nasal swab samples. Red boxes highlight the individual signals.

**Table S3. Primer sequences for loop-mediated isothermal amplification of RSV strain A2 RNA.**

| **Primer** | **Sequence (5′-3′)** |
| --- | --- |
| F3 | GCTGTTCAATACAATGTCCTAGA |
| B3 | GGTAAATTTGCTGGGCATT |
| FIP | TCTGCTGGCATGGATGATTGGAGACGATGATCCTGCATCA |
| BIP | CTAGTGAAACAAATATCCACACCCAGCACTGCACTTCTTGAGTT |
| LF | ACATGGGCACCCATATTGTAAG |
| LB | AGGGACCTTCATTAAGAGTCATGAT |

*A 10x primer mix contains 16 μM FIP/BIP, 2 μM F3/B3, and 4 μM LF/LB.


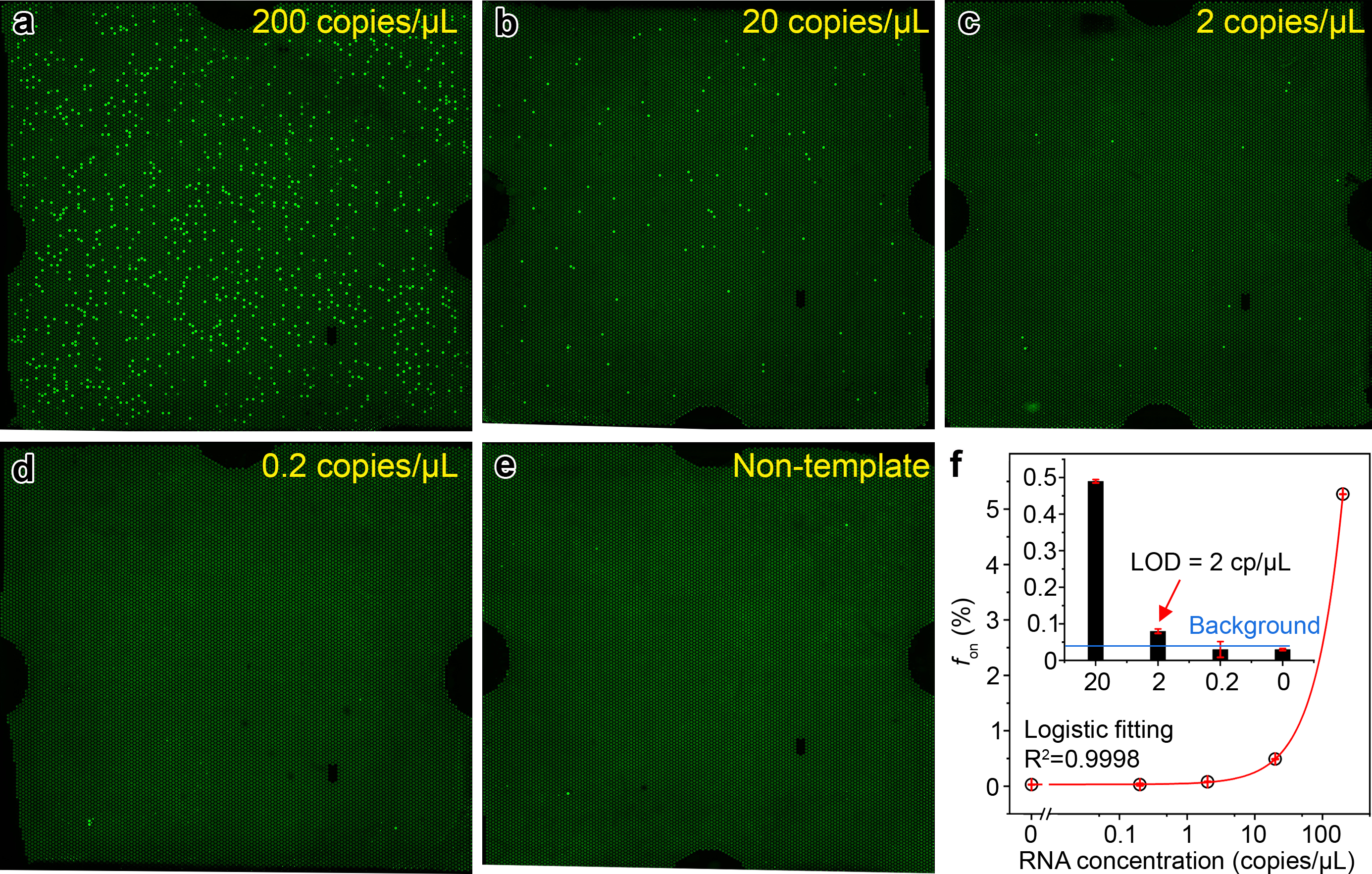


Fig. S16. Detection of RSV RNA via digital loop-mediated isothermal amplification (dLAMP). (a-e) Fluorescence images of the microwell chips after dLAMP with varied RNA inputs. All images have the intensity range of 0-10,000 relative fluorescence units (RFU). (f) A plot of the frequency of positive wells (*f*_on_) against different RNA inputs (copies per microliter). The positive wells were determined as the maximal fluorescence intensity greater than a threshold of three times standard deviations above the mean of the negative control*.* Inset shows the limit of detection (LOD), where the background is set as three times standard deviation above the zero calibrator. Each well of the microfluidic chip has a volume of 750 pL.


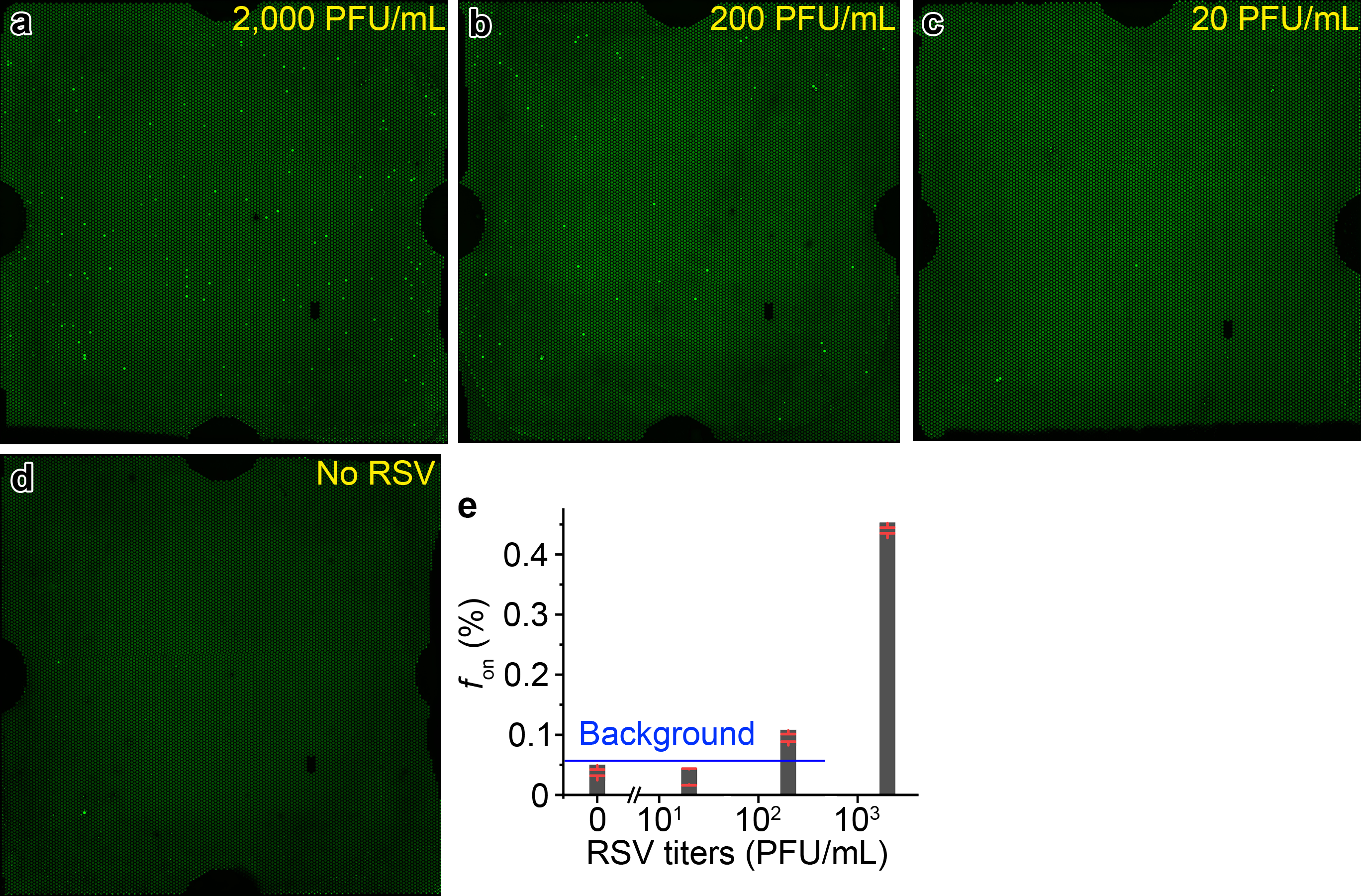


Fig. S17. Detection of RNA extracts from RSV spiked samples via dLAMP. (a-d) Fluorescence images of the microwell chips after dLAMP with varied RSV inputs. All images have the intensity range of 0-10,000 RFU. (e) A plot of *f*_on_ against RSV titers. The background is set as 3-times standard deviation above the zero calibrator.

Note: According to the calibration curve from Fig. S15f, we can use the background-subtracted frequency (*f*_on_’) to calculate the concentration of RNA detected in RSV extracts. The results are provided below. Based on the results, we estimated that 1 PFU/mL RSV has roughly 0.01 copies/ µL RNA extracts.

| Spiked RSV  (PFU/mL) | *f*_on_’ | RNA conc.  (copies/µL) |
| --- | --- | --- |
| 200 | 0.058 | 2.2 |
| 2,000 | 0.403 | 15.3 |


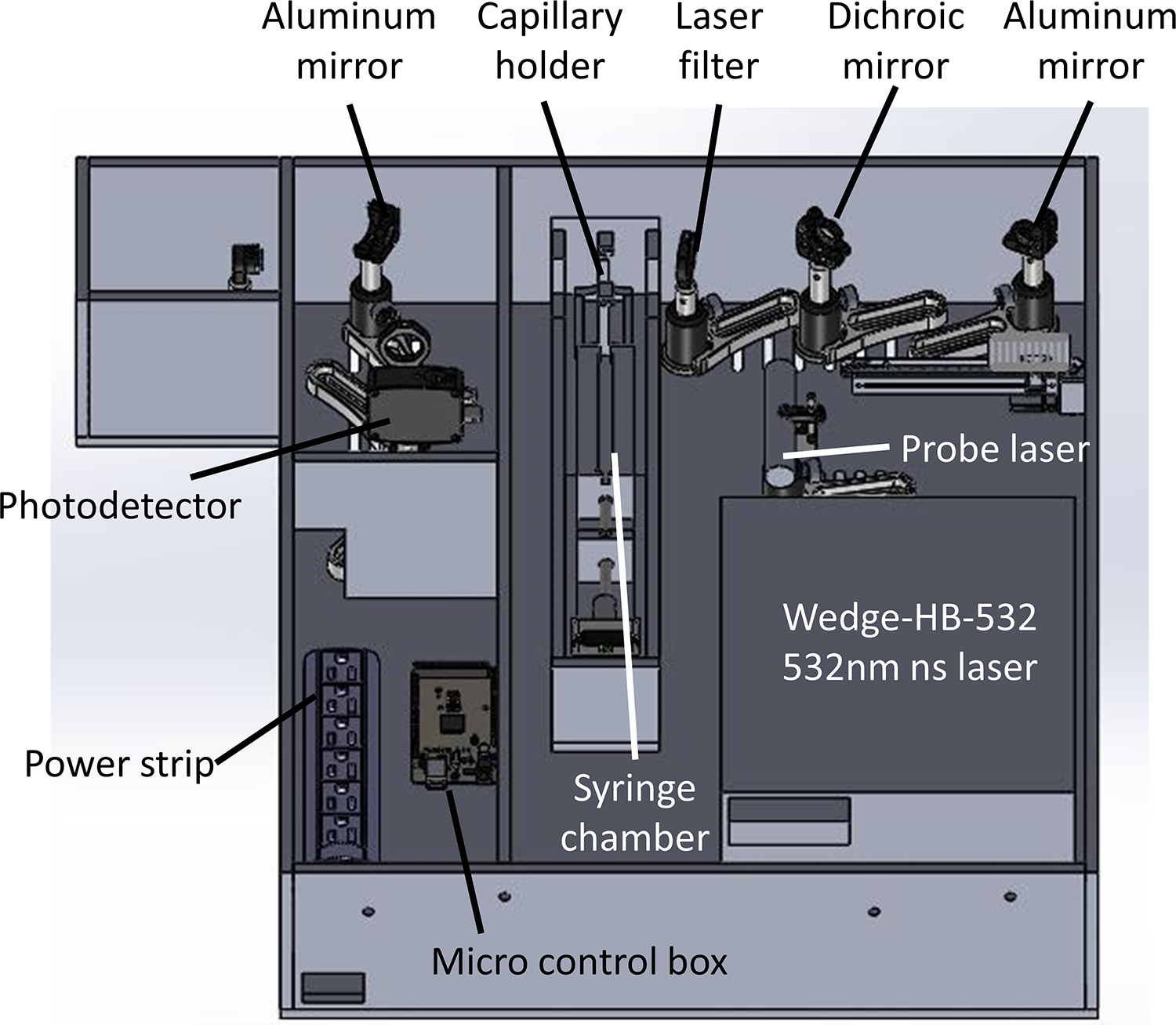


Fig. S18. Design of benchtop device integrating DIAMOND. The device has a dimension of 15×15×6 inches.


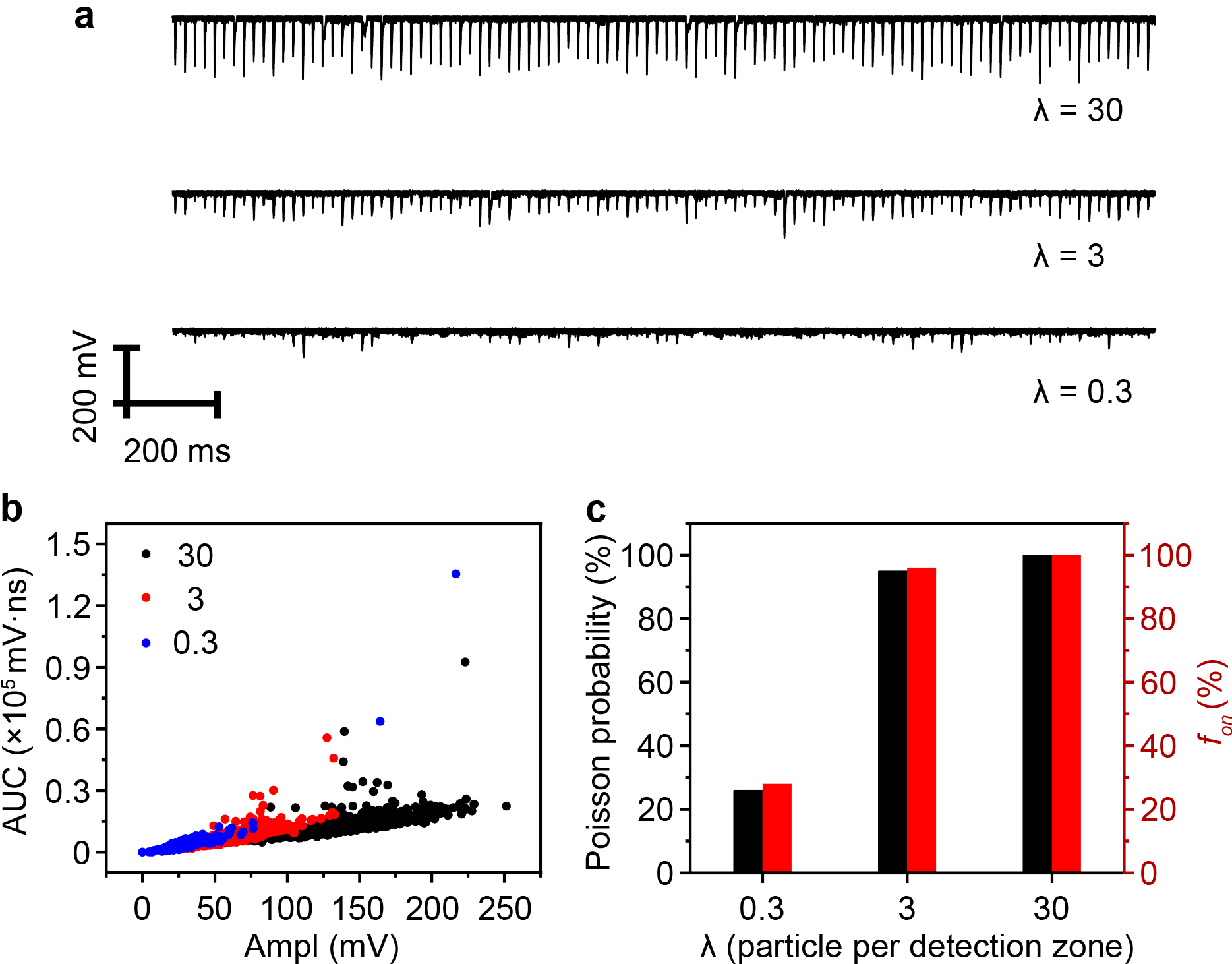


Fig. S19. Detection of 75 nm AuNPs by DIAMOND using a nanosecond laser (Wedge-HB-532, RPMC). (a) Representative PNB signal traces (100 pulses) for 75 nm AuNP suspensions with different particle concentrations. (b) Bivariate plots of amplitude and AUC were extracted from 3,000 pulses for the three samples in (a). (c) Bar plot of experimental frequencies (*f*_on_) as determined in (b) and theoretical probability predicted by Poisson statistics for the given λ.


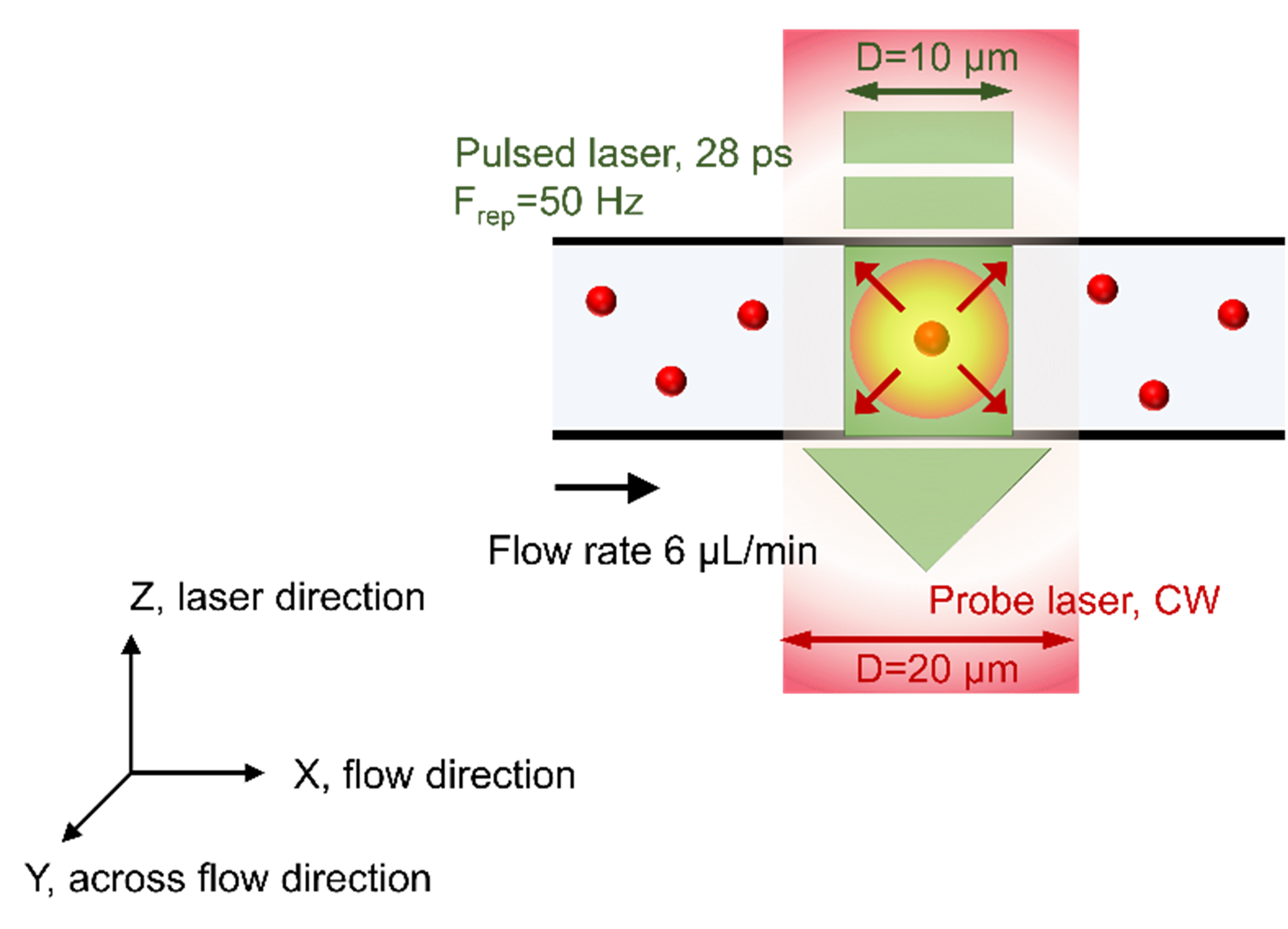


Fig. S20. The schematic illustrates the calculation of sampling efficiency for the probe beam based on the current setup.

Note: Considering that the beam size of the pump laser is 10 µm, we calculated its sampling efficiency as half to the probe laser, equaling 20% and 5% along and across the flow direction, respectively. See the calculation of sampling efficiency for the probe beam below.

$$\mathbf{Sampling efficiency along the flow=}\frac{\mathbf{Speed of flow passing through the laser}}{\mathbf{Speed of flow by syringe pump}}\mathbf{=}\frac{\boldsymbol{20 \mu m \times50 Hz}}{\frac{\boldsymbol{6 \mu L\cdot}\boldsymbol{min}^{\boldsymbol{-1}}}{{\boldsymbol{200}\boldsymbol{\mu m}}^{\boldsymbol{2}}}}\boldsymbol{\times}\boldsymbol{100\%=40\%}$$

$$\mathbf{Sampling efficiency across the flow=}\frac{\mathbf{Beam diameter}}{\mathbf{Capillary size}}\mathbf{=}\frac{\boldsymbol{20 \mu m}}{\boldsymbol{200 \mu m}}\boldsymbol{\times100\%=10\%}$$




Fig. S21. The detection performance for the detection of 75 nm AuNPs (λ=0.0004) by DIAMOND with increasing counting number.

Table S4. A prediction on the sensitivity enhancement by increasing the counting number for DIAMOND.

| ^a^Expected particle number (λ) | Poisson prediction (%) | Average positive counts of 1 thousand counts | Average positive counts of 1 million counts |
| --- | --- | --- | --- |
| 0.1 | 9.5 | 95 | 95,000 |
| 0.01 | 1 | 10^*^ | 10,000 |
| 0.001 | 0.1 | 1 | 1,0002 |
| 0.0001 | 0.01 | 0.1 | 100 |
| 0.00001 | 0.001 | 0.01 | 10^*^ |
| 0.000001 | 0.0001 | 0.001 | 1 |

^a^: λ is proportional to the target concentration for a given detection volume

^*^: estimated LOD


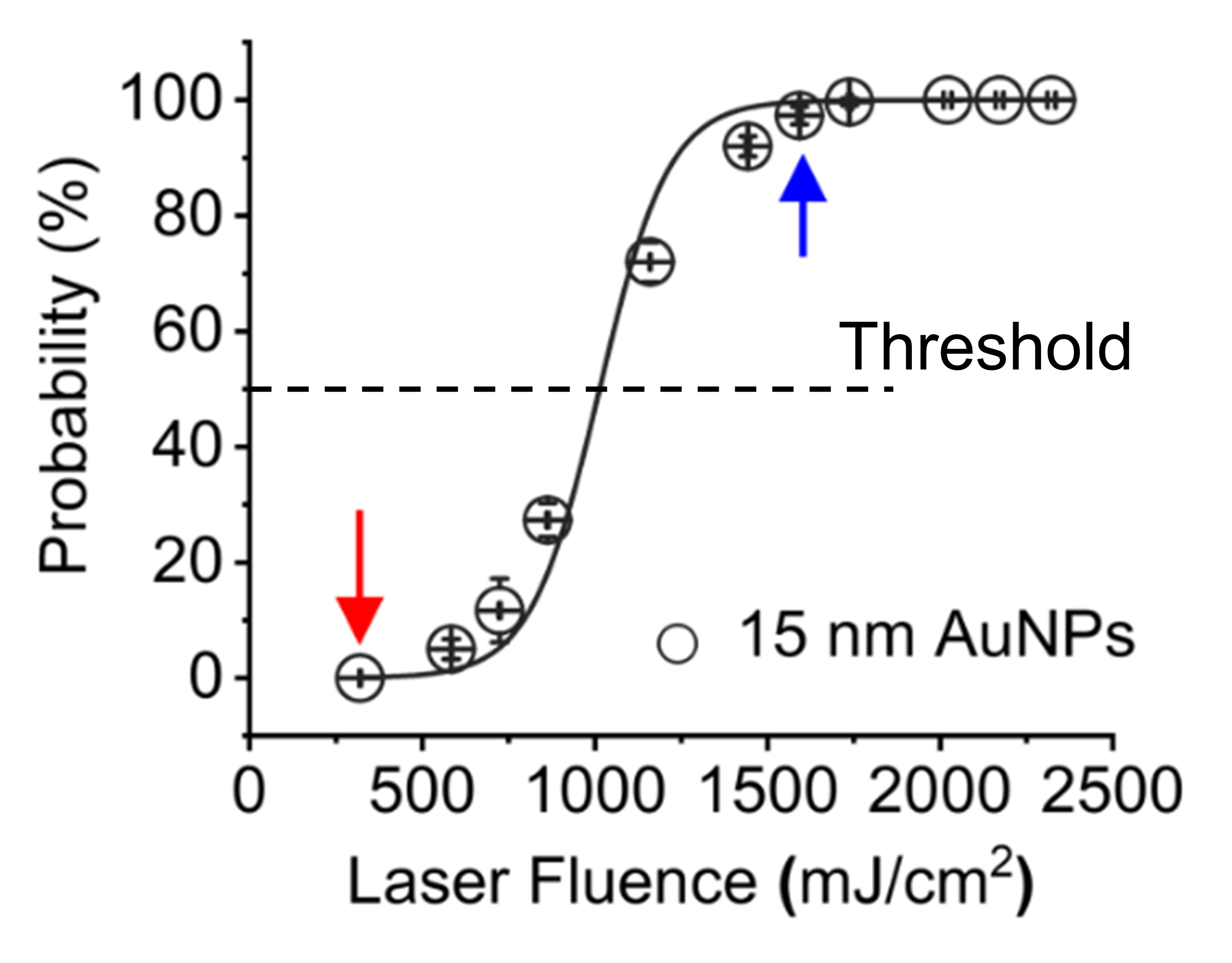


Fig. S22. Example of the PNB generation probability curve of 15 nm AuNPs used to determine the laser fluence threshold. The laser fluence corresponds to that of 50% probability (dashed line) is set as the threshold. The red arrow marks the laser fluence for 0 probability, while the blue arrow marks the laser fluence for 100% probability.

**
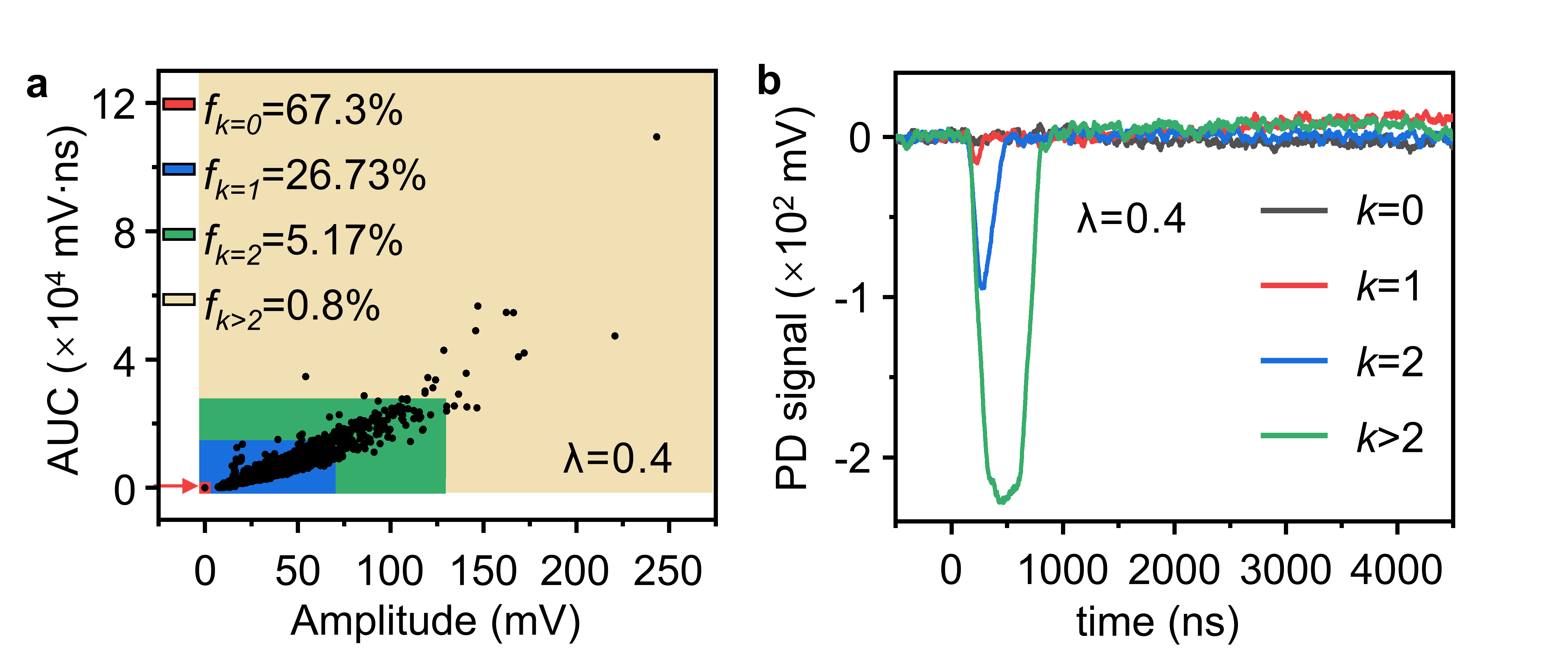
**

**Fig. S23. Data sorting by** **a gating method.** (a) A bivariate plot of amplitude and AUC extracted from 3,000 pulses for 75 nm AuNPs with λ=0.4 as shown in **Fig. 2a**. The Poisson probabilities are used to determine thresholds (T=*μ*+nꞏ*σ*, *μ* is mean value, *σ* is standard deviation, n>0) as highlighted by the colors. The frequency (*f*) of scatters in each color background is counted and shown. Red arrow highlights the scatters with zero amplitude and area-under-curve (AUC). (b) Representative PNB signals of none, single and multiple AuNPs that were extracted from (a) in different color regions. PD is photodetector.

Table S5. One-pot recipe for the synthesis of AuNPs based on the seed-growth method.

| Reagent  Size (nm) | H_2_O  *mL* | HAuCl_4_  *25 mM, mL* | Na_3_CA·2H_2_O  *15 mM, mL* | 15 nm Au Seeds  *2.23 nM, mL* | Hydroquinone  *25 mM, mL* |
| --- | --- | --- | --- | --- | --- |
| 35 | 84.84 | 0.875 | 0.875 | 12.533 | 0.875 |
| 50 | 93.39 | 0.963 | 0.963 | 3.714 | 0.963 |
| 70 | 96.67 | 0.997 | 0.997 | 0.338 | 0.997 |


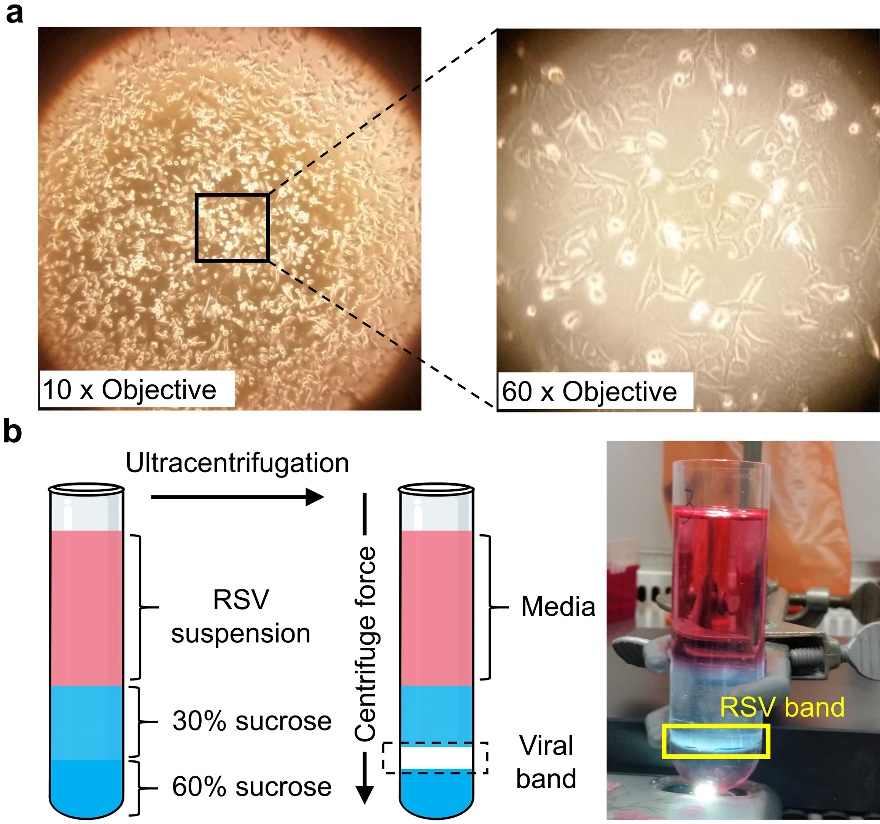


Fig. S24. Large scale preparation of RSV A2. (a) Photographs of clinical RSV A2 strain infected HEp-2 cells at day 4. (b) Sucrose density gradient for RSV purification.


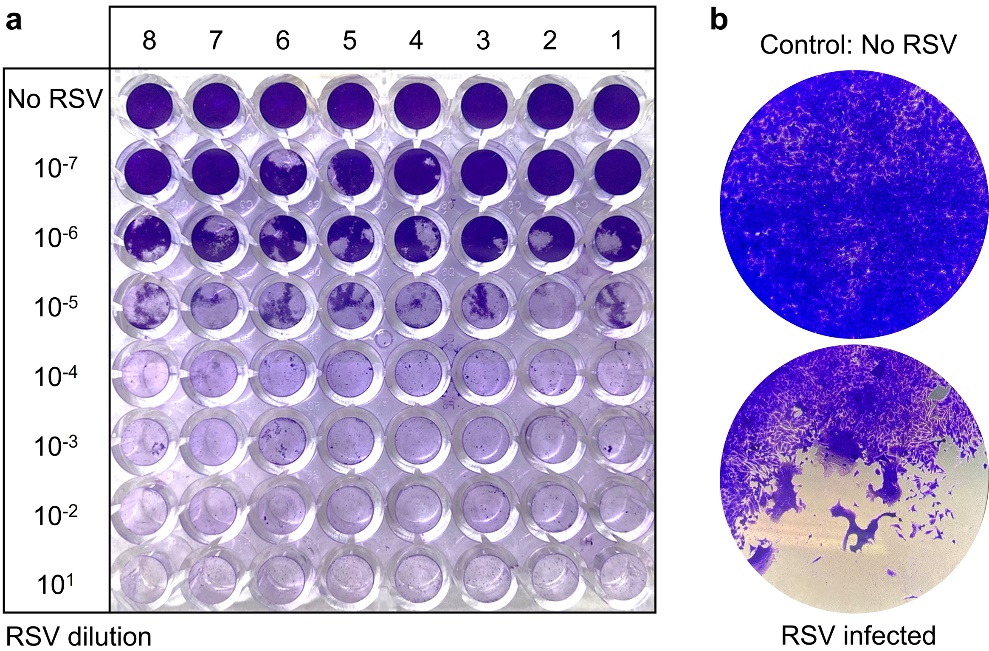


Fig. S25. Endpoint dilution assay for RSV quantification. (a) 96 well plate layout for serial dilutions of RSV stock inoculated onto HEp-2 cells. (b) Example of control well and RSV infected well after staining with crystal violet dye.
